# Top 100 Cited Articles on Clinical Hematopoietic Stem Cell Transplantation: A Bibliometric Analysis

**DOI:** 10.3389/fmed.2022.872692

**Published:** 2022-06-06

**Authors:** Moazzam Shahzad, Sibgha Gull Chaudhary, Iqra Anwar, Muhammad Arslan, Zehra Naseem, Naira T. Fatima, Sakina Abbas, Tayyaba Ali, Raheel S. Siddiqui, Peiman Hematti, Sunil H. Abhyankar, Joseph P. McGuirk, Muhammad Umair Mushtaq

**Affiliations:** ^1^Division of Hematologic Malignancies & Cellular Therapeutics, University of Kansas Medical Center, Kansas City, MO, United States; ^2^Department of Medicine, St Mary's Medical Center, Huntington, WV, United States; ^3^Division of Hematology/Oncology, University of Wisconsin School of Medicine & Public Health, Madison, WI, United States

**Keywords:** hematopoietic stem cell transplant, bibliometric analysis, citation classics, Hematology, Oncology

## Abstract

We conducted a bibliometric analysis to identify scholarly impact and factors associated with the top 100 cited articles on clinical hematopoietic stem cell transplantation (HSCT). In January 2021, a title-specific search was conducted. Non-HSCT and pre-clinical (*in-vitro* and animal) studies were excluded. A total of 39,406 records were identified and a list of the top 100 articles was made. Articles included in our study were characterized by the citations received, publication year, topic, study design, authors, h-index, and institutions. Linear regression analyses were performed. The 100 most cited articles were published over 52 years from 1968 to 2020, with a maximum number of articles (*n* = 40) published in the 1990s decade. Top-100 articles were cited 62,002 times with a median citation count of 465 (range 336–2240). The top-cited articles originated from 12 countries. United States contributed 69 articles. The University of Washington Fred Hutchinson Cancer Center (*n* = 15) was the leading institution. Blood (*n* = 32) and New England Journal of Medicine (*n* = 31) made the greatest contribution, and 52 manuscripts were clinical trials. The first author's H-index significantly correlated with citation count while journal impact factor, years since publication, first author's gender, and the number of authors did not have a significant association with the number of citations. In a multivariate regression model, the first author's h-index (regression coefficient 5.46, 95% confidence interval 2.99 to 7.93, *p* < 0.001) independently correlated with the citation count. Our study highlights the most influential articles on clinical HSCT and provides valuable insight for future research needs of the specialty.

## Background

Hematopoietic stem-cell transplantation (HSCT), the most widely used cellular immunotherapy, was first performed by E. Donnell Thomas in 1957 as a new form of treatment for hematologic malignancies and has evolved into an adoptive immune therapy for malignant and non-malignant hematologic and immune disorders with a potential for cure (1). As a potentially lifesaving treatment, HSCT not only requires careful selection of stem-cell donors, recipients, and conditioning regimens but also close monitoring and aggressive management of clinical complications (2).

Bibliometric analysis constitutes the determination and evaluation of impact of research and scholarly publications with the help of techniques like the analysis of citations to assess the effect of a particular manuscript over a specified period of time (3). This serves as a mode to evaluate educational achievement for authors, as the number of citations a paper receives is usually proportional to the influence of that paper in a particular discipline (3). Large data sets can be examined by bibliometric methods and can help decision making regarding individuals, institutions, and organization. Citations are less susceptible to manipulation and are a reliable indicator (4). Since 1987, bibliometric analyses have been published on various fields of medicine (5); however, a bibliometric analysis of top-cited articles on HSCT is yet to be done. In this study, we conducted a bibliometric analysis to evaluate, identify, and characterize the 100 top-cited articles on HSCT to highlight the current state-of-the-art of the specialty and identify directions for future clinical research.

## Methods

### Data Sources and Search Strategy

The Clarivate Analytics Web of Science core collection (WoS) was accessed in January 2021 and a title-specific search was conducted using the MeSh terms and keywords “hematopoietic stem-cell transplantation,” “transplantation, hematopoietic stem cell,” “stem cell transplantation, hematopoietic,” “bone-marrow transplantation,” “transplantation, bone marrow,” “grafting, bone marrow,” “bone marrow grafting,” “transplantation bone marrow cell,” “bone marrow cell transplantation,” “stem cell transplant,” “allogeneic hematopoietic stem cell transplant,” “autologous hematopoietic stem cell transplant.” A total of 39,406 records were identified (6).

### Data Extraction

A list of the top 100 cited articles was compiled and arranged in descending order according to the WoS number of citations. Data were collected for study title, study design, study population, study topic, number of citations, journal name, journal impact factor (IF), and number of authors. The study design was classified as review, meta-analysis, retrospective, prospective, randomized/non-randomized clinical trials, and guidelines/consensus. Clinical studies in HSCT were included in the analysis. Non-HSCT and pre-clinical (*in-vitro* and animal) studies were excluded. The primary and corresponding author's information included: author name, gender, affiliations, and h-indices. If the corresponding author was the last author, then primary author's information was used. Data were extracted by four authors (MA, ZN, NF, and SA) independently to ensure accuracy. The extracted data were double-checked by MS. and MM.

### Data Analysis

Data were analyzed using SPSS version 21. Descriptive statistics were used to describe characteristics of 100 top-cited articles. Top 100-cited articles were categorized by year. Factors associated with five or more manuscripts among top-100 cited articles were explored including the journals, first authors' characteristics (institution, country, H-index), and study details (type and topic). Linear regression analysis was performed and regression coefficients (R) with 95% confidence intervals (CI) were obtained. In univariate and multivariate linear regression analyses, number of citations received by an article were compared with gender, years since publication, journal IF, and the number of authors. Statistical significance was considered at *p* < 0.05.

## Results

**Figure 1:**
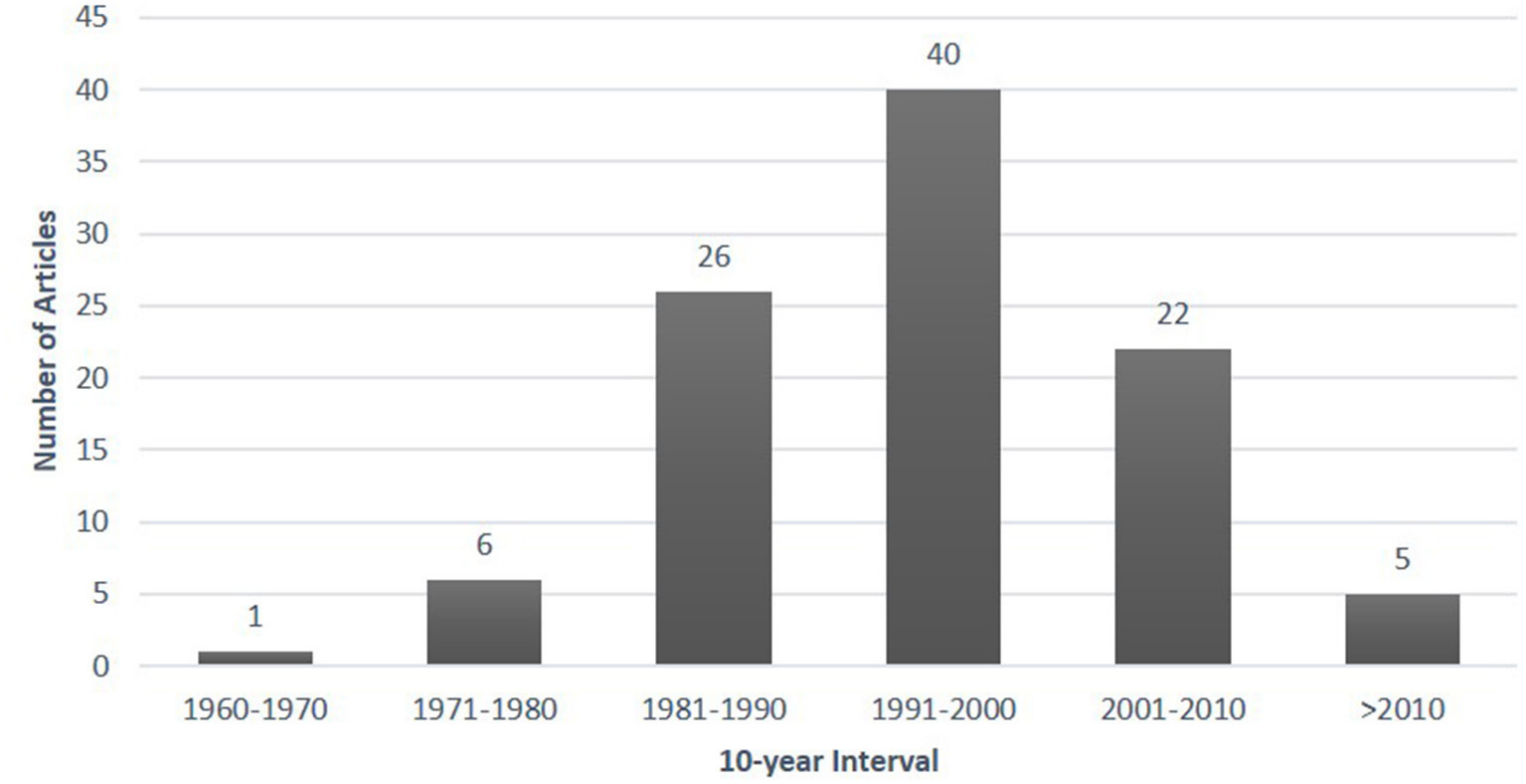
The 100 top-cited articles on hematopoietic stem cell transplantation by 10-year intervals.

The 100 most cited articles in the field of HSCT were included (Supplementary Table 1). Top-100 articles were cited 62,002 times with a median citation count of 465 (range 334–2240). Among the top 100, only nine articles were cited more than 1000 times, while three articles were cited more than 2000 times. The 100 most cited articles in the field were published over 52 years from 1968 to 2020. The 10-year interval from 1991–2000 had the highest number of publications (*n* = 40) (Figure 1). The years 1988 and 1999 had the greatest number of publications accounting for seven manuscripts each.

Blood (*n* = 32) and New England Journal of Medicine (*n* = 31) had the highest number of publications. The top-cited articles originated from 12 countries and the United States (US) contributed 69 articles. Bone marrow transplantation was the most discussed topic (*n* = 21), and a clinical trial was the most common manuscript type (*n* = 52). The University of Washington Fred Hutchinson Cancer Center (*n* = 15) was the leading institution. Thirteen authors had five or more manuscripts in the top-100 articles, and Rainer Strob, MD had the highest number of publications (*n* = 12) (Table 1).

**Table 1:** Factors associated with five or more articles in the 100 top-cited articles on hematopoietic stem cell transplantation.

**Factor**	**Articles in top 100 (*n*)**	**Citations (*n*)**
**Journal (impact factor)**		
Blood (17.8)	32	17400
New England Journal of Medicine (78)	31	24061
The Lancet (88.4)	9	4762
Clinical Infectious Diseases (13)	5	3301
Annals of Internal Medicine (13.2)	5	3065
Journal of Clinical Oncology (32.96)	5	3009
**Country**		
United States	69	42412
United Kingdom	10	5226
France	7	6697
**Institution**		
Fred Hutchinson Cancer Research Center (US)	15	8489
Memorial Sloan-Kettering Cancer Center (US)	8	3712
Johns Hopkins University School of Medicine (US)	7	4058
University of Washington School of Medicine (US)	5	5238
Medical College of Wisconsin (US)	5	4407
University of Minnesota, Minneapolis (US)	5	2659
**Authors (H-index)**		
Storb R.F (149)	12	8759
Thomas E.D (119)	9	7618
Gale R.P (107)	7	5227
Buckner C.D (100)	7	6547
Weisdorf D.J (114)	7	3499
Horowitz M.M (116)	7	5283
Martin P.J (118)	6	2954
Deeg H.J (106)	6	2859
Appelbaum F.R (153)	6	3656
Witherspoon R.P (70)	6	2556
Oreilly R.J (75)	6	2925
Clift R.A (89)	6	6500
Wingard J.R (92)	5	3018
**Type of Article**		
Clinical trial	52	32790
Retrospective cohort study	22	12448
Prospective cohort study	13	6726
Review study	9	7912
**Topic**		
Bone marrow transplantation	21	138891
Autologous bone marrow transplantation	13	7799
Fungal infection	8	5852
Allogeneic bone marrow transplantation	7	2826
Graft versus host disease	6	2884

In univariate linear regression analyses, the first author's h-index (R 5.42, 95% CI 3.11 to 7.73, *p* < 0.001) correlated with the citation count while, years since publication (R −4.62, 95% CI −13.78 to 4.54, *p* = 0.319), journal impact factor (R 2.48, 95% CI −0.57 to 5.53, *p* = 0.109), first author's gender (R 83.72, 95% CI −148.76 to 316.21, *p* = 0.477) and the number of authors (R 1.33, 95% CI −13.18 to 15.85, *p* = 0.856) did not have a significant association with the number of citations. In multi-variate linear regression analysis adjusted for years since publication, journal impact factor, first author's gender and the number of authors, the first author's h-index (R 5.46, 95% CI 2.99 to 7.93, *p* < 0.001) remained an independent predictor of the citation count.

## Discussion

Our study highlights the 100 most cited articles on hematopoietic stem cell transplantation. Clinical trials were the main study type with a peak increase in publications between 1991 to 2000. The scarcity of studies before the 1990s demonstrates recent advancements in HSCT. Previous bibliometric analysis has demonstrated the highest contributions to stem cell research were made in the past two decades (7). Although the first human bone marrow transfusion was given to a patient suffering from aplastic anemia in 1939 (8), our findings revealed that the majority of the highly cited papers were not cited until 1968, in contrast to previous bibliometric analysis in neurosurgery (9).

Blood, the official journal of the American Society of Hematology, and the New England Journal of Medicine were the top-cited journals. Although authors centralize their publications in journals with wider readership and higher impact factors, we did not find a significant correlation between the impact factor of a journal and the number of citations it received. The US contributed a major number of publications similar to a previous study in stem cell laboratory research but in contrast, our study has a minimal contribution from Asia (7).

There are several limitations of our analysis. While categorizing articles in descending order according to the number of citations, older manuscripts were higher in the top 100 as compared to recent publications. The reason could be that the latest publications are cited fewer times during the years preceding their publication. We only used WoS for our literature search and took the top 100 WoS cited articles that can be different from other citation databases; however, WoS is considered a standard citation database for medical research as well as more accurate in document type labelling (10).

## Conclusion

To our knowledge, this is the first study to identify the 100 most cited papers on clinical HSCT. Our study highlights themost influential articles on HSCT, centralization of research to North American and European institutions, and provides valuable insight for future research needs of the specialty.

## Data Availability Statement

The original contributions presented in the study are included in the article/Supplementary Material, further inquiries can be directed to the corresponding author/s.

## Author Contributions

All authors contributed to the manuscript and fulfilled criteria per the uniform requirements set forth by the International Committee of Medical Journal Editors (ICJME) guidelines. All authors contributed to the article and approved the submitted version.

## Conflict of Interest

The authors declare that the research was conducted in the absence of any commercial or financial relationships that could be construed as a potential conflict of interest.

## Publisher's Note

All claims expressed in this article are solely those of the authors and do not necessarily represent those of their affiliated organizations, or those of the publisher, the editors and the reviewers. Any product that may be evaluated in this article, or claim that may be made by its manufacturer, is not guaranteed or endorsed by the publisher.
