# Supplementary material for: Top 100 Cited Articles on Clinical Hematopoietic Stem Cell Transplantation: A Bibliometric Analysis
Source: Front Med (Lausanne). 2022 Jun 6;9:872692. doi: 10.3389/fmed.2022.872692 (PMC9208296; doi:10.3389/fmed.2022.872692)
Supplement: Supplementary file 1 [file Table_1.docx]

**Supplemental Table 1: Top 100 cited articles on hematopoietic stem cell transplantation**

| First Author | **Title** | **Year** | **Journal** | **Citations (n)** | **Country** | **Study Design** | **Study Topic** |
| --- | --- | --- | --- | --- | --- | --- | --- |
| Horowitz, MM | Graft-versus-leukemia reactions after bone marrow transplantation. (PMID2297567) | 1990 | Blood | 2240 | USA | Clinical Trial | Graft versus Leukemia reaction |
| Thomas, ED | Bone-marrow transplantation (first of two parts) *. (PMID234595) | 1975 | N. Engl. J. Med. | 2238 | USA | Review | Bone-marrow Transplantation. |
| Attal, M | A prospective, randomized trial of autologous bone marrow transplantation and chemotherapy in multiple myeloma. Intergroupe Français du Myélome. (PMID8649495) | 1996 | N. Engl. J. Med. | 2176 | France | Clinical Trial | Multiple myeloma treatment |
| Slavin, S | Nonmyeloablative stem cell transplantation and cell therapy as an alternative to conventional bone marrow transplantation with lethal cytoreduction for the treatment of malignant and nonmalignant hematologic diseases. (PMID9446633) | 1998 | Blood | 1692 | Israel | Clinical Trial | Nonmyeloablative stem cell Transplantation |
| Thomas, ED | Bone-marrow transplantation (second of two parts) *. (PMID235092) | 1975 | N. Engl. J. Med. | 1692 | USA | Review | Bone-marrow Transplantation. |
| Philip, T | Autologous bone marrow transplantation as compared with salvage chemotherapy in relapses of chemotherapy-sensitive non-Hodgkin's lymphoma. (PMID7477169) | 1995 | N. Engl. J. Med. | 1673 | France | Clinical Trial | Autologous Bone-marrow Transplantation |
| Matthay, KK | Treatment of high-risk neuroblastoma with intensive chemotherapy, radiotherapy, autologous bone marrow transplantation, and 13-cis-retinoic acid. Children's Cancer Group. (PMID10519894) | 1999 | N. Engl. J. Med. | 1315 | USA | Clinical Trial | High-risk Neuroblastoma treatment |
| Copelan, EA | Hematopoietic stem-cell transplantation*. (PMID16641398) | 2006 | N. Engl. J. Med. | 1276 | USA | Review | Hematopoietic Stem-cell Transplantation |
| Marr, KA | Epidemiology and outcome of mould infections in hematopoietic stem cell transplant recipients. (PMID11880955) | 2002 | Clin. Infect. Dis. | 1042 | USA | Retrospective | Fungal Infection |
| Collins, RH | Donor leukocyte infusions in 140 patients with relapsed malignancy after allogeneic bone marrow transplantation. (PMID9053463) | 1997 | J. Clin. Oncol. | 962 | USA | Retrospective | Donor leukocyte infusion |
| Goodman, JR | A controlled trial of fluconazole to prevent fungal infections in patients undergoing bone marrow transplantation. (PMID1542320) | 1992 | N. Engl. J. Med. | 937 | USA | Clinical Trial | Fungal Infection |
| Brenner, MK | Gene-marking to trace origin of relapse after autologous bone-marrow transplantation. (PMID8093407) | 1993 | Lancet | 890 | USA | Case Reports | Autologous Bone-marrow Transplantation |
| Luznik, L | HLA-haploidentical bone marrow transplantation for hematologic malignancies using nonmyeloablative conditioning and high-dose, post-transplantation cyclophosphamide. (PMID18489989) | 2008 | Biol. Blood Marrow Transplant. | 886 | USA | Clinical Trial | Haploidentical Bone marrow transplantation |
| Mcdonald, GB | Veno-occlusive disease of the liver and multiorgan failure after bone marrow transplantation: a cohort study of 355 patients. (PMID8420443) | 1993 | Ann. Intern. Med. | 883 | USA | Prospective Cohort | Veno-occlusive disease |
| Goldman, JM | Bone marrow transplantation for chronic myelogenous leukemia in chronic phase. Increased risk for relapse associated with T-cell depletion. (PMID3285744) | 1988 | Ann. Intern. Med. | 854 | USA | Retrospective | Bone Marrow Transplantation |
| Kontoyiannis, DP | Prospective surveillance for invasive fungal infections in hematopoietic stem cell transplant recipients, 2001-2006: overview of the Transplant-Associated Infection Surveillance Network (TRANSNET) Database. (PMID20218877) | 2010 | Clin. Infect. Dis. | 832 | USA | Prospective | Fungal Infection |
| Papadopoulos, EB | Infusions of donor leukocytes to treat Epstein-Barr virus-associated lymphoproliferative disorders after allogeneic bone marrow transplantation. (PMID8093146) | 1994 | N. Engl. J. Med. | 805 | USA | Clinical Trial | Donor Leukocytes Infusion |
| Brandt, SJ | Effect of recombinant human granulocyte-macrophage colony-stimulating factor on hematopoietic reconstitution after high-dose chemotherapy and autologous bone marrow transplantation. (PMID3281007) | 1988 | N. Engl. J. Med. | 803 | England | Clinical Trial | Hematopoietic Reconstitution |
| Wingard, JR | Increase in Candida krusei infection among patients with bone marrow transplantation and neutropenia treated prophylactically with fluconazole. (PMID1669837) | 1991 | N. Engl. J. Med. | 800 | USA | Retrospective | Fungal infection |
| Bearman, SI | Regimen-related toxicity in patients undergoing bone marrow transplantation. (PMID3049951) | 1988 | J. Clin. Oncol. | 800 | USA | Retrospective | Bone Marrow Transplantion |
| Thierry Philip | High-dose therapy and autologous bone marrow transplantation after failure of conventional chemotherapy in adults with intermediate-grade or high-grade non-Hodgkin's lymphoma. (PMID3295541) | 1987 | N. Engl. J. Med. | 794 | France | Clinical Trial | Intermediate-High grade non-Hodgkin’s lymphoma treatment |
| Mcdonald, GB | Veno-occlusive disease of the liver after bone marrow transplantation: diagnosis, incidence, and predisposing factors. (PMID6363247) | 1984 | Hepatology | 739 | USA | Prospective | Veno-occlusive disease |
| Linch, DC | Dose intensification with autologous bone-marrow transplantation in relapsed and resistant Hodgkin's disease: results of a BNLI randomized trial. (PMID8096958) | 1993 | Lancet | 739 | UK | Clinical Trial | Autologous bone-marrow transplantation |
| Jones, RJ | Veno-occlusive disease of the liver following bone marrow transplantation. (PMID3321587) | 1987 | Transplantation | 720 | USA | Prospective | Veno-occlusive disease |
| Zittoun, R | Autologous or allogeneic bone marrow transplantation compared with intensive chemotherapy in acute myelogenous leukemia. European Organization for Research and Treatment of Cancer (EORTC) and the Gruppo Italiano Malattie Ematologiche Maligne dell'Adulto (GIMEMA) Leukemia Cooperative Groups. (PMID7808487) | 1995 | N. Engl. J. Med. | 711 | France | Clinical Trial | Acute Myeloid Leukemia treatment |
|  | Immunologic purging of marrow assessed by PCR before autologous bone marrow transplantation for B-cell lymphoma. (PMID1944436) | 1991 | N. Engl. J. Med. | 689 | UK | Clinical Trial | Autologous bone marrow transplantation |
| Ruggeri, L | Role of natural killer cell alloreactivity in HLA-mismatched hematopoietic stem cell transplantation. (PMID10381530) | 1999 | Blood | 686 | Italy | Clinical Trial | Natural Killer Cell |
| Marr, KA | Invasive aspergillosis in allogeneic stem cell transplant recipients: changes in epidemiology and risk factors. (PMID12393425) | 2002 | Blood | 673 | USA | Retrospective | Fungal Infection |
| Curtis, RE | Solid cancers after bone marrow transplantation. (PMID9070469) | 1997 | N. Engl. J. Med. | 631 | USA | Retrospective | Bone marrow transplantation |
| Mackinnon, S | Adoptive immunotherapy evaluating escalating doses of donor leukocytes for relapse of chronic myeloid leukemia after bone marrow transplantation: separation of graft-versus-leukemia responses from graft-versus-host disease. (PMID7632930) | 1995 | Blood | 614 | USA | Clinical Trial | Donor Leukocyte Infusion |
| Holler, E | Increased serum levels of tumor necrosis factor alpha precede major complications of bone marrow transplantation. (PMID2405918) | 1990 | Blood | 592 | Germany | Retrospective | Bone marrow transplantation |
| James, A | Bone marrow transplantation*. (PMID8114836) | 1994 | N. Engl. J. Med. | 582 | USA | Review | Bone marrow transplantation |
| Sullivan, KM | Chronic graft-versus-host disease and other late complications of bone marrow transplantation*. (PMID1887253) | 1991 | Semin. Hematol. | 580 | USA | Review | Graft versus Host Disease |
| Tutschka, PJ | Bone marrow transplantation for leukemia following a new busulfan and cyclophosphamide regimen. (PMID3311203) | 1987 | Blood | 575 | USA | Clinical Trial | Bone marrow Transplantation |
| Reusser, P | Cytotoxic T-lymphocyte response to cytomegalovirus after human allogeneic bone marrow transplantation: pattern of recovery and correlation with cytomegalovirus infection and disease. (PMID1652311) | 1991 | Blood | 559 | USA | Clinical Trial | Cytomegalovirus infection |
| Ziegler, TR | Clinical and metabolic efficacy of glutamine-supplemented parenteral nutrition after bone marrow transplantation. A randomized, double-blind, controlled study. (PMID1567096) | 1992 | Ann. Intern. Med. | 552 | USA | Clinical Trial | Parental Nutrition |
| Wald, A | Epidemiology of Aspergillus infections in a large cohort of patients undergoing bone marrow transplantation. (PMID9180187) | 1997 | J. Exp. Med. | 548 | USA | Retrospective | Fungal Infection |
| Schmitz, N | Randomized trial of filgrastim-mobilized peripheral blood progenitor cell transplantation versus autologous bone-marrow transplantation in lymphoma patients. (PMID8598700) | 1996 | Lancet | 542 | Germany | Clinical Trial | Autologous bone-marrow transplantation |
| Lucarelli, G | Bone marrow transplantation in patients with thalassemia. (PMID2300104) | 1990 | N. Engl. J. Med. | 541 | Italy | Clinical Trial | Bone marrow transplantation |
| Sullivan, KM | Influence of acute and chronic graft-versus-host disease on relapse and survival after bone marrow transplantation from HLA-identical siblings as treatment of acute and chronic leukemia. (PMID2653460) | 1989 | Blood | 540 | USA | Retrospective | Graft versus Host Disease |
| Goodrich, JM | Early treatment with ganciclovir to prevent cytomegalovirus disease after allogeneic bone marrow transplantation. (PMID1658652) | 1991 | N. Engl. J. Med. | 529 | USA | Clinical Trial | Cytomegalovirus Infection |
| Powles, RL | Cyclosporin A to prevent graft-versus-host disease in man after allogeneic bone-marrow transplantation. (PMID6101787) | 1980 | Lancet | 526 | UK | Clinical Trial | Graft versus Host Disease |
| Neofytos, D. | Epidemiology and outcome of invasive fungal infection in adult hematopoietic stem cell transplant recipients: analysis of Multicenter Prospective Antifungal Therapy (PATH) Alliance registry. (PMID19115967) | 2009 | Clin. Infect. Dis. | 518 | USA | Prospective | Fungal Infection |
| Yeager, AM | Autologous bone marrow transplantation in patients with acute nonlymphocytic leukemia, using ex vivo marrow treatment with 4-hydroperoxycyclophosphamide. (PMID3523241) | 1986 | N. Engl. J. Med. | 513 | USA | Clinical Trial | Autologous bone marrow transplantation |
| Aversa, F | Full haplotype-mismatched hematopoietic stem-cell transplantation: a phase II study in patients with acute leukemia at high risk of relapse. (PMID15753458) | 2005 | J. Clin. Oncol. | 509 | France | Clinical Trial | Haploidentical stem-cell transplantation |
| Van Burik, JAH | Micafungin versus fluconazole for prophylaxis against invasive fungal infections during neutropenia in patients undergoing hematopoietic stem cell transplantation. (PMID15546073) | 2004 | Clin. Infect. Dis. | 502 | USA | Clinical Trial | Fungal Infection |
| Flomenberg, N | Impact of HLA class I and class II high-resolution matching on outcomes of unrelated donor bone marrow transplantation: HLA-C mismatching is associated with a strong adverse effect on transplantation outcome. (PMID15191952) | 2004 | Blood | 493 | USA | Retrospective | HLA matching |
| Buckley, RH | Hematopoietic stem-cell transplantation for the treatment of severe combined immunodeficiency. (PMID10021471) | 1999 | N. Engl. J. Med. | 491 | England | Clinical Trial | Hematopoietic Stem-Cell Transplantation |
| Socie, G | Long-term survival and late deaths after allogeneic bone marrow transplantation. Late Effects Working Committee of the International Bone Marrow Transplant Registry. (PMID10387937) | 1999 | Blood | 490 | France | Retrospective | Allogeneic Bone Marrow Transplantation |
| Nemunaitis, J | Recombinant granulocyte-macrophage colony-stimulating factor after autologous bone marrow transplantation for lymphoid cancer. (PMID1903847) | 1991 | N. Engl. J. Med. | 465 | USA | Clinical Trial | Autologous Bone Marrow Transplantation |
| Sanders, JE | Pregnancies following high-dose cyclophosphamide with or without high-dose busulfan or total-body irradiation and bone marrow transplantation. (PMID8639928) | 1996 | N. Engl. J. Med. | 465 | USA | Retrospective | Bone marrow transplantation |
| Camitta, BM | A prospective study of androgens and bone marrow transplantation for treatment of severe aplastic anemia. (PMID32941) | 1979 | Blood | 462 | USA | Prospective | Severe Aplastic Anemia treatment |
| Shapiro, RS | Epstein-Barr virus associated B cell lymphoproliferative disorders following bone marrow transplantation. (PMID2833957) | 1988 | Blood | 461 | USA | Retrospective | Lymphoproliferative disorders |
| Cassileth, PA | Chemotherapy compared with autologous or allogeneic bone marrow transplantation in the management of acute myeloid leukemia in first remission. (PMID9834301) | 1998 | Lancet | 459 | USA | Clinical Trial | Acute Myeloid Leukemia treatment |
| Henter, JI | Treatment of hemophagocytic lymphohistiocytosis with HLH-94 immunochemotherapy and bone marrow transplantation. (PMID12239144) | 2002 | Blood | 457 | Sweden | Clinical Trial | hemophagocytic lymphohistiocytosis Treatment |
| Sheridan, WP | Granulocyte colony-stimulating factor and neutrophil recovery after high-dose chemotherapy and autologous bone marrow transplantation. (PMID2477656) | 1989 | Lancet | 457 | Austrailia | Clinical Trial | Autologous bone marrow transplantation |
| Curtis, RE | Risk of lymphoproliferative disorders after bone marrow transplantation: a multi-institutional study. (PMID10498590) | 1999 | N. Engl. J. Med. | 443 | USA | Retrospective | Lymphoproliferative disorders |
| Winston, DJ | Infectious complications of human bone marrow transplantation*. (PMID368507) | 1979 | Medicine | 437 | USA | Review | Infectious complications |
| Goulmy, E | Mismatches of minor histocompatibility antigens between HLA-identical donors and recipients and the development of graft-versus-host disease after bone marrow transplantation. (PMID8532022) | 1996 | N. Engl. J. Med. | 434 | Netherland | Clinical Trial | Graft versus Host Disease |
| Burnett, AK | Randomized comparison of addition of autologous bone-marrow transplantation to intensive chemotherapy for acute myeloid leukemia in first remission: results of MRC AML 10 trial. UK Medical Research Council Adult and Children's Leukemia Working Parties. (PMID9504514) | 1998 | N. Engl. J. Med. | 433 | UK | Clinical Trial | Acute Myeloid Leukemia treatment |
| Bach, FH | Bone-marrow transplantation in a patient with the Wiskott-Aldrich syndrome. (PMID4177931) | 1968 | Lancet | 433 | USA | Case Report | Bone-marrow transplantation |
| Coccia, PF | Successful bone-marrow transplantation for infantile malignant osteopetrosis. (PMID6986555) | 1980 | N. Engl. J. Med. | 424 | USA | Case Report | Bone-marrow transplantation |
| Mielcarek, M | Graft-versus-host disease after nonmyeloablative versus conventional hematopoietic stem cell transplantation. (PMID12663454) | 2003 | Blood | 421 | USA | Retrospective | Graft versus Host Disease |
| Oreilly, RJ | Allogenic bone marrow transplantation: current status and future directions*. (PMID6354307) | 1983 | Blood | 421 | USA | Review | Allogenic bone marrow transplantation |
| Kessinger, A | Autologous peripheral hematopoietic stem cell transplantation restores hematopoietic function following marrow ablative therapy. (PMID2894230) | 1988 | Blood | 419 | USA | Clinical Trial | Autologous bone-marrow transplantation |
| Cutler, CS | A decision analysis of allogeneic bone marrow transplantation for the myelodysplastic syndromes: delayed transplantation for low-risk myelodysplasia is associated with improved outcome. (PMID15039286) | 2004 | Blood | 418 | USA | Retrospective | Allogeneic bone marrow transplantation |
| Goldman, JM | Bone marrow transplantation for patients with chronic myeloid leukemia. (PMID3510388) | 1986 | N. Engl. J. Med. | 411 | UK | Clinical Trail | Bone marrow transplantation |
| Taur, Y | Intestinal domination and the risk of bacteremia in patients undergoing allogeneic hematopoietic stem cell transplantation. (PMID22718773) | 2012 | Clin. Infect. Dis. | 407 | USA | Prospective | Allogeneic hematopoietic stem cell transplantation |
| Gianni, AM | High-dose chemotherapy and autologous bone marrow transplantation compared with MACOP-B in aggressive B-cell lymphoma. (PMID9113932) | 1997 | N. Engl. J. Med. | 403 | Italy | Clinical Trial | Aggressive B-cell lymphoma treatment |
| Emanuel, D | Cytomegalovirus pneumonia after bone marrow transplantation successfully treated with the combination of ganciclovir and high-dose intravenous immune globulin. (PMID2847609) | 1988 | Ann. Intern. Med. | 403 | USA | Clinical Trial | Cytomegalovirus Infection |
| Giebel, S | Survival advantage with KIR ligand incompatibility in hematopoietic stem cell transplantation from unrelated donors. (PMID12689936) | 2003 | Blood | 402 | Poland | Clinical Trial | KIR ligand incompatibility |
| Sonis, ST | Oral mucositis and the clinical and economic outcomes of hematopoietic stem-cell transplantation. (PMID11304772) | 2001 | J. Clin. Oncol. | 398 | USA | Prospective | Hematopoietic stem cell transplantation |
| Horwitz, EM | Clinical responses to bone marrow transplantation in children with severe osteogenesis imperfecta. (PMID11222364) | 2001 | Blood | 396 | USA | Clinical Trial | Bone marrow transplantation |
| Cobbold, M | Adoptive transfer of cytomegalovirus-specific CTL to stem cell transplant patients after selection by HLA-peptide tetramers. (PMID16061727) | 2005 | J. Exp. Med. | 388 | UK | Clinical Trial | Cytomegalovirus specific T-Lymphocytes transfusion |
| Ratanatharathorn, V | Phase III study comparing methotrexate and tacrolimus (prograf, FK506) with methotrexate and cyclosporine for graft-versus-host disease prophylaxis after HLA-identical sibling bone marrow transplantation. (PMID9746768) | 1998 | Blood | 383 | USA | Clinical Trial | Graft versus Host Disease |
| Walters, MC | Bone marrow transplantation for sickle cell disease. (PMID8663884) | 1996 | Blood | 383 | USA | Clinical Trial | Bone marrow transplantation |
| Taur, Y; Jenq | The effects of intestinal tract bacterial diversity on mortality following allogeneic hematopoietic stem cell transplantation. (PMID24939656) | 2014 | Blood | 380 | USA | Prospective | Allogeneic hematopoietic stem cell transplantation |
| Hobbs, JR | Reversal of clinical features of Hurler's disease and biochemical improvement after treatment by bone-marrow transplantation. (PMID6116856) | 1981 | Lancet | 379 | UK | Case Report | Bone-marrow transplantation |
| Zutter, MM | Epstein-Barr virus lymphoproliferation after bone marrow transplantation. (PMID2840986) | 1988 | Blood | 378 | USA | Retrospective | Epstein-Barr virus lymphoproliferation |
| Weiner, RS | Interstitial pneumonitis after bone marrow transplantation. Assessment of risk factors. (PMID3511812) | 1986 | Ann. Intern. Med. | 373 | USA | Retrospective | Interstitial pneumonitis |
| Einsele, H | Polymerase chain reaction monitoring reduces the incidence of cytomegalovirus disease and the duration and side effects of antiviral therapy after bone marrow transplantation. (PMID7670117) | 1995 | Blood | 373 | Germany | Clinical Trial | Cytomegalovirus Infection |
| Ball, LM | Cotransplantation of ex vivo expanded mesenchymal stem cells accelerates lymphocyte recovery and may reduce the risk of graft failure in haploidentical hematopoietic stem-cell transplantation. (PMID17638847) | 2007 | Blood | 372 | Netherland | Clinical Trial | Haploidentical stem-cell transplantation |
| Voltarelli, JC | Autologous nonmyeloablative hematopoietic stem cell transplantation in newly diagnosed type 1 diabetes mellitus. (PMID17426276) | 2007 | JAMA-J. Am. Med. Assoc. | 367 | USA | Clinical Trial | Autologous bone marrow transplantation |
| Cross, NCP | Competitive polymerase chain reaction to estimate the number of BCR-ABL transcripts in chronic myeloid leukemia patients after bone marrow transplantation. (PMID8400243) | 1999 | Blood | 367 | UK | Clinical Trial | Bone marrow transplantation |
| Slavin, S | Allogeneic cell therapy with donor peripheral blood cells and recombinant human interleukin-2 to treat leukemia relapse after allogeneic bone marrow transplantation. (PMID8630379) | 1996 | Blood | 364 | Israel | Prospective | Allogeneic bone marrow transplantation |
| Witherspoon, RP | Secondary cancers after bone marrow transplantation for leukemia or aplastic anemia. (PMID2671734) | 1989 | N. Engl. J. Med. | 362 | USA | Retrospective | Secondary Cancers |
| Rill, DR | Direct demonstration that autologous bone marrow transplantation for solid tumors can return a multiplicity of tumorigenic cells. (PMID8025266) | 1994 | Blood | 361 | USA | Clinical Trial | Autologous bone marrow transplantation |
| Gratwohl, A | Hematopoietic stem cell transplantation: a global perspective. (PMID20424252) | 2010 | JAMA-J. Am. Med. Assoc. | 359 | USA | Retrospective | Hematopoietic stem cell transplantation |
| Maertens, J | European Guidelines for Antifungal Management in Leukemia and Hematopoietic Stem Cell Transplant Recipients: Summary of The Ecil 3-2009 Update*. (PMID20661235) | 2010 | Bone Marrow Transplant. | 352 | Belgium | Review | Antifungal management |
| Grochow, LB | Pharmacokinetics of busulfan: correlation with veno-occlusive disease in patients undergoing bone marrow transplantation. (PMID2591002) | 1989 | Cancer Chemother. Pharmacol. | 350 | USA | Clinical Trial | Veno-Occlusive Disease |
| Keith MS | Immunomodulatory and antimicrobial efficacy of intravenous immunoglobulin in bone marrow transplantation. (PMID2167452) | 1990 | N. Engl. J. Med. | 347 | USA | Clinical Trial | Intravenous Immunoglobulin |
| Kochenderfer, JN | Donor-derived CD19-targeted T cells cause regression of malignancy persisting after allogeneic hematopoietic stem cell transplantation. (PMID24055823) | 2020 | Blood | 346 | USA | Clinical Trial | Allogeneic hematopoietic stem cell transplantation |
| Hernigou, P | Treatment of osteonecrosis with autologous bone marrow grafting. (PMID12461352) | 2002 | Clin. Orthop. Rel. Res. | 344 | France | Prospective | Autologous bone marrow grafting |
| Small, TN | Comparison of immune reconstitution after unrelated and related T-cell-depleted bone marrow transplantation: effect of patient age and donor leukocyte infusions. (PMID9885208) | 1999 | Blood | 344 | USA | Prospective | Immune Reconstitution |
| Anthony FS | Adenovirus infections in patients undergoing bone-marrow transplantation. (PMID2982098) | 1985 | N. Engl. J. Med. | 343 | USA | Retrospective | Adenovirus Infection |
| Armand, PA | Disabling immune tolerance by programmed death-1 blockade with pidilizumab after autologous hematopoietic stem-cell transplantation for diffuse large B-cell lymphoma: results of an international phase II trial. (PMID24127452) | 2013 | J. Clin. Oncol. | 340 | USA | Clinical Trial | Autologous hematopoietic stem-cell transplantation |
| Katharine C | Improved outcome in HLA-identical sibling hematopoietic stem-cell transplantation for acute myelogenous leukemia predicted by KIR and HLA genotypes. (PMID15731175) | 2005 | Blood | 338 | USA | Clinical Trial | Haploidentical stem-cell transplantation |
| Durie, BGM | Bortezomib with lenalidomide and dexamethasone versus lenalidomide and dexamethasone alone in patients with newly diagnosed myeloma without intent for immediate autologous stem-cell transplant (SWOG S0777): a randomised, open-label, phase 3 trial. (PMID28017406) | 2017 | Lancet | 337 | USA | Clinical Trial | Multiple Myeloma Treatment |
| KERSEY, JH | Comparison of autologous and allogeneic bone marrow transplantation for treatment of high-risk refractory acute lymphoblastic leukemia. (PMID3302708) | 1987 | N. Engl. J. Med. | 335 | USA | Prospective | Acute Lymphoblastic Leukemia |
| Haynes, BF | The role of the thymus in immune reconstitution in aging, bone marrow transplantation, and HIV-1 infection*. (PMID10837068) | 2000 | Annu. Rev. Immunol. | 334 | USA | Review | Immune Reconstitution |
| *Review Studies | | | | | |  |  |
